# Supplementary material for: Coordinated social interactions are supported by integrated neural representations
Source: Soc Cogn Affect Neurosci. 2024 Dec 6;19(1):nsae089. doi: 10.1093/scan/nsae089 (PMC11642603; doi:10.1093/scan/nsae089)
Supplement: nsae089_Supp [file nsae089_supp.zip › nsae089_Supp/scan-24-104-File009.docx]

| Social Context | Contrast | Mean (±sd) | BF_10_ distribution < 0.5 | t | p-value | Cohen’s d |
| --- | --- | --- | --- | --- | --- | --- |
| Joint | CD vs DC | 0.50 (±0.01) | 0.88 | 1.40 | 0.08 | 0.23 |
|  | CC vs DD | 0.50 (±0.01) | 0.36 | 0.07 | 0.47 | 0.01 |
|  | Own: C vs D | 0.50 (±0.01) | 0.80 | -1.32 | 0.90 | 0.22 |
|  | Partner: C vs D | 0.50 (±0.01) | 0.55 | -0.96 | 0.83 | 0.16 |
| Parallel | CD vs DC | 0.50 (± 0.01) | 0.98 | 1.49 | 0.07 | 0.24 |
|  | CC vs DD | 0.50 (± 0.01) | 0.46 | -0.73 | 0.76 | 0.12 |
|  | Own: C vs D | 0.50 (±0.01) | 1.09 | 1.57 | 0.06 | 0.26 |
|  | Partner: C vs D | 0.50 (±0.01) | 0.37 | 0.31 | 0.38 | 0.05 |
