# Supplementary material for: Coordinated social interactions are supported by integrated neural representations
Source: Soc Cogn Affect Neurosci. 2024 Dec 6;19(1):nsae089. doi: 10.1093/scan/nsae089 (PMC11642603; doi:10.1093/scan/nsae089)
Supplement: nsae089_Supp [file nsae089_supp.zip › nsae089_Supp/Supplementary Materials.docx]

**Supplementary Materials**

1. *Null distributions of classification accuracies*

To test whether each of the observed classification accuracies distribution was significantly above chance, we computed null distributions by randomly permuting the classification labels. This procedure yielded a null distribution for each of the contrasts of interest. None of the null distributions was significantly different from the theoretical chance level of 50% (i.e., 0.5), as resulting from one-tailed one-sample t-test. The distributions are depicted in Figure S1 and their statistical details are reported in Table S1.

1. *Control Analysis on VMI and Drawing Time*

We fitted an additional model including the centered factor Drawing Time and its interactions with the factors Social context and Congruency (Figure S2). The rationale of fitting this model was accounting for a potential effect of the speed of drawing on the distortion in the trajectories. While the effects of Social context, Congruency and their interaction were unaffected by the inclusion of the factor Drawing time, we found a significant interaction of Congruency and Drawing Time (β = -0.78, 95% CI = [-1.27, -0.30], t_16625_ = -3.18, *p* = 0.001). This interaction indicates that slower movements are associated with larger distortions in incongruent trials, discarding the possibility of a speed-accuracy trade-off between movement duration and variability in drawn trajectories.

**Table S1**

*Null distributions descriptive statistics and tests against theoretical chance (50%)*

| Social Context | Contrast | Mean (±sd) | BF_10_ distribution < 0.5 | t | p-value | Cohen’s d |
| --- | --- | --- | --- | --- | --- | --- |
| Joint | CD vs DC | 0.50 (±0.01) | 0.88 | 1.40 | 0.08 | 0.23 |
|  | CC vs DD | 0.50 (±0.01) | 0.36 | 0.07 | 0.47 | 0.01 |
|  | Own: C vs D | 0.50 (±0.01) | 0.80 | -1.32 | 0.90 | 0.22 |
|  | Partner: C vs D | 0.50 (±0.01) | 0.55 | -0.96 | 0.83 | 0.16 |
| Parallel | CD vs DC | 0.50 (± 0.01) | 0.98 | 1.49 | 0.07 | 0.24 |
|  | CC vs DD | 0.50 (± 0.01) | 0.46 | -0.73 | 0.76 | 0.12 |
|  | Own: C vs D | 0.50 (±0.01) | 1.09 | 1.57 | 0.06 | 0.26 |
|  | Partner: C vs D | 0.50 (±0.01) | 0.37 | 0.31 | 0.38 | 0.05 |

**Figure S1**

*Null distributions for each contrast of interest*

**
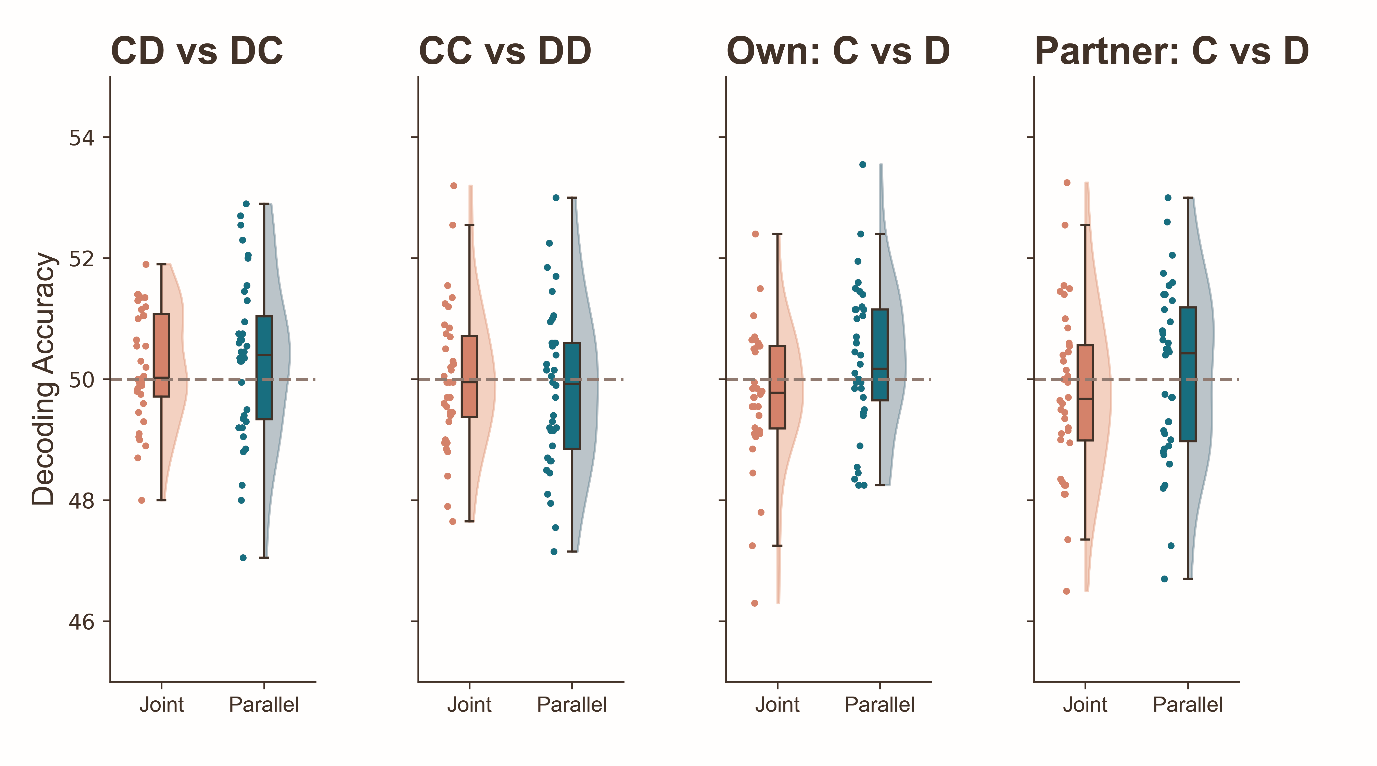
**

**Figure S2**

*Control Analysis on VMI and Drawing Time*

*
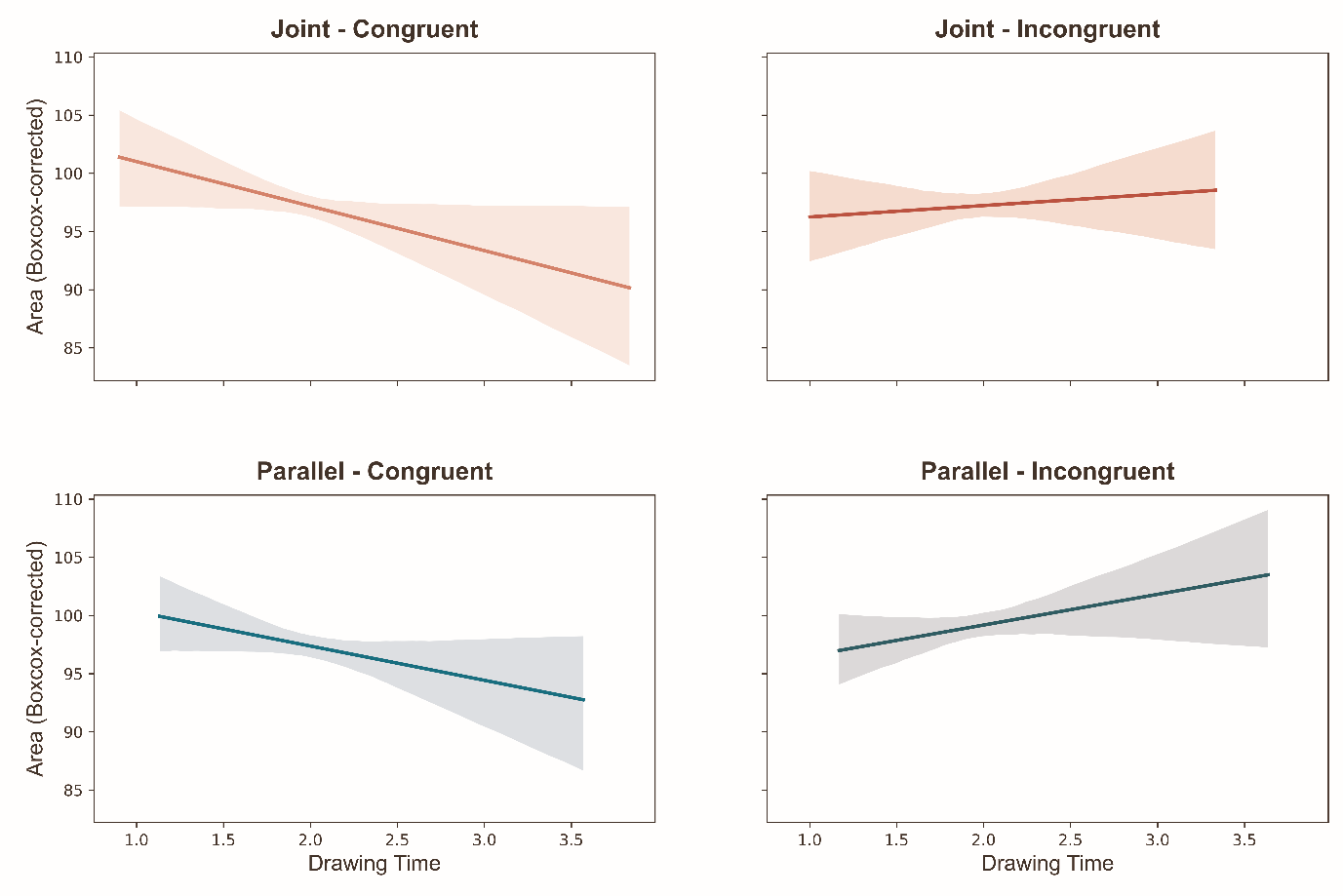
*

*Note*. The significant interaction of the factor Congruency and Drawing Time suggests that larger VMI is observed in incongruent trials with longer Drawing Time, as opposed to congruent trials. This indicates that a phenomenon akin to a ‘speed-accuracy trade-off’ (i.e., poorer performance in favor of faster execution) can be observed only in congruent but not in incongruent trials.
