# Supplementary figures and images for: Coordinated social interactions are supported by integrated neural representations
Source: Soc Cogn Affect Neurosci. 2024 Dec 6;19(1):nsae089. doi: 10.1093/scan/nsae089 (PMC11642603; doi:10.1093/scan/nsae089)

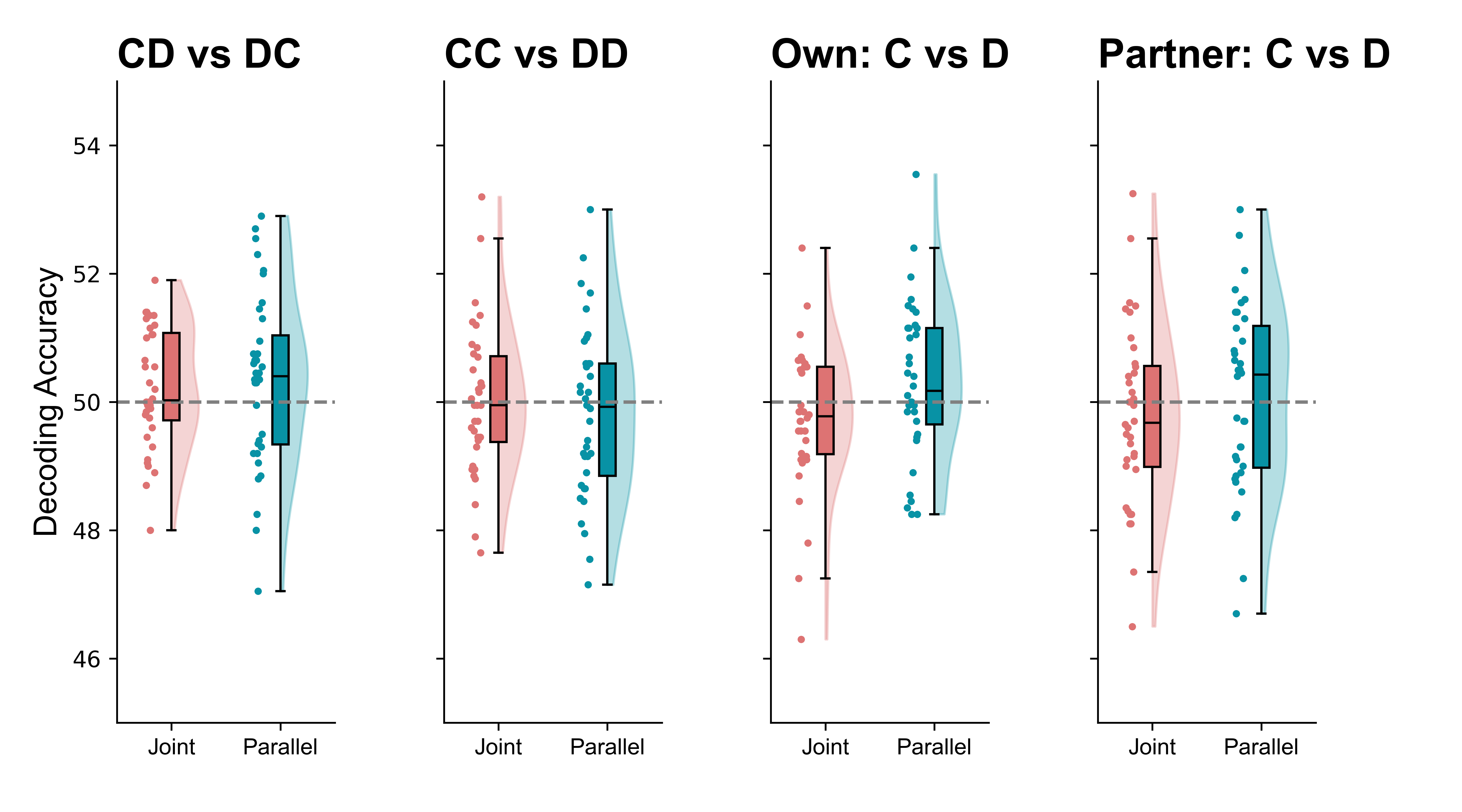

Supplement: nsae089_Supp [file nsae089_supp.zip › nsae089_Supp/scan-24-104-File010.tiff]
